# Clinical significance of Phosphatidyl Inositol Synthase overexpression in oral cancer

**DOI:** 10.1186/1471-2407-10-168

**Published:** 2010-04-28

**Authors:** Jatinder Kaur, Meenakshi Sawhney, Siddartha DattaGupta, Nootan K Shukla, Anurag Srivastava, Ranju Ralhan

**Affiliations:** 1 Department of Biochemistry, All India Institute for Medical Sciences, Ansari Nagar, New Delhi -110029, India; 2 Department of Pathology, All India Institute for Medical Sciences, Ansari Nagar, New Delhi -110029, India; 3 Department of Surgical Oncology, Institute of Rotary Cancer Hospital, All India Institute for Medical Sciences, Ansari Nagar, New Delhi -110029, India; 4 Department of Surgical Disciplines, All India Institute for Medical Sciences, Ansari Nagar, New Delhi -110029, India; 5 Joseph & Mildred Sonshine Family Centre for Head & Neck Disease, Mount Sinai Hospital; 6 Department of Otolaryngology-Head and Neck Surgery, Mount Sinai Hospital; 7 Department of Pathology and Laboratory Medicine, Mount Sinai Hospital, Joseph & Wolf Lebovic Health Complex, 600 University Avenue, Room 6-500, Toronto, Ontario, Canada M5G 1X5; 8 Department of Otolaryngology-Head and Neck Surgery, University of Toronto; Toronto, Ontario, Canada M5G 2N2; 9 Department of Chemistry and Centre for Research in Mass Spectrometry, CB- Room 246, York University, 4700 Keele Street, Toronto, Ontario, Canada M3J 1P3

## Abstract

**Background:**

We reported increased levels of Phosphatidyl Inositol synthase (PI synthase), (enzyme that catalyses phosphatidyl inositol (PI) synthesis-implicated in intracellular signaling and regulation of cell growth) in smokeless tobacco (ST) exposed oral cell cultures by differential display. This study determined the clinical significance of PI synthase overexpression in oral squamous cell carcinoma (OSCC) and premalignant lesions (leukoplakia), and identified the downstream signaling proteins in PI synthase pathway that are perturbed by smokeless tobacco (ST) exposure.

**Methods:**

Tissue microarray (TMA) Immunohistochemistry, Western blotting, Confocal laser scan microscopy, RT-PCR were performed to define the expression of PI synthase in clinical samples and in oral cell culture systems.

**Results:**

Significant increase in PI synthase immunoreactivity was observed in premalignant lesions and OSCCs as compared to oral normal tissues (p = 0.000). Further, PI synthase expression was significantly associated with de-differentiation of OSCCs, (p = 0.005) and tobacco consumption (p = 0.03, OR = 9.0). Exposure of oral cell systems to smokeless tobacco (ST) in vitro confirmed increase in PI synthase, Phosphatidylinositol 3-kinase (PI3K) and cyclin D1 levels.

**Conclusion:**

Collectively, increased PI synthase expression was found to be an early event in oral cancer and a target for smokeless tobacco.

## Background

Five percent of all cancers occur in the head and neck, with over 500,000 cases reported annually worldwide, and mortality rate of about 50% [1-3]; approximately half of these occur in the oral cavity [4]. Head-and-neck cancer sites are readily amenable to clinical examination, yet a lack of suitable molecular markers for early detection and risk assessment is clearly reflected by the fact that more than 50% of all oral squamous cell carcinoma (OSCC) patients have advanced disease at the time of diagnosis [1,3,5,6]. Indeed, the five-year survival rates of OSCC patients are in general poor (about 50% overall) and the prognosis of advanced OSCC cases has not improved much over the past three decades [3,5]. Epidemiological evidence shows a correlation between use of smokeless tobacco (ST) and lesions of the oral cavity as well as with incidence of oral cancer [7-9]. OSCCs are often preceded by clinically evident oral lesions (OLs), often leukoplakia, and the risk of multiple cancers is 5-10 times greater in patients with OSCCs preceded by leukoplakia [10]. These OLs are reported to be more common in chewing tobacco related oral cancer in India [11].

Intense efforts are being directed towards developing accurate predictors of clinical outcome using high throughput techniques such as differential display-reverse transcription PCR (DD), cDNA microarrays and proteomics to assess global gene/protein expression patterns in head and neck cancer [12-15]. In search of such novel molecular targets, our laboratory reported increased levels of phosphatidyl inositol synthase (PI Synthase) or CDP-diacylglycerol-inositol 3-phosphatidyl transferase (CDIPT) transcripts in cell cultures from a human oral lesion (AMOL), exposed to ST extracts using DD [16], providing the rationale for in-depth investigation of biological and clinical significance of its expression in oral cancer.

PI Synthase (PIS) (EC 2.7.8.11) is a 24-kDa membrane-bound enzyme, which catalyzes the last step in the de novo biosynthesis of phosphatidylinositol (PI) by catalyzing the condensation of CDP-diacylglycerol and myo-inositol to form PI and CMP. PI is involved in protein membrane anchoring, and is the precursor for the second messengers- inositol-tri-phosphate and diacylglycerol (DG). These ubiquitous second messengers function downstream of many G protein-coupled receptors and tyrosine kinases regulating cell growth, calcium metabolism, and PKC activity. The biological role of PI is of considerable interest due to the involvement of PI and its phosphorylated derivatives in intracellular signal transduction. Phosphatidylinositol 3-kinase (PI3K) catalyses the phosphorylation of PI in the 3-OH position of the inositol ring. The PI3K pathway regulates various cellular processes, such as proliferation, growth, apoptosis and cytoskeletal rearrangement [17,18].

Herein, we determined the effect of ST on the expression PI Synthase and its downstream targets PI3K and cyclinD1 in oral cell systems. Further, we investigated the clinical significance of PI Synthase expression in oral cancer using immunohistochemistry.

## Methods

### Cell culture

Human head and neck squamous carcinoma cell lines, HSC2, SCC-4 and cell culture from an oral lesion (OL), AMOL [19], were grown in monolayer cultures in Dulbecco's modified eagle medium (DMEM) and Ham F-12 (DMEM-F12) (Sigma, MO) supplemented with 10% fetal bovine serum (FBS), 2.5 mM L-glutamine, 1× sodium pyruvate (supplied as 100× stock with a concentration of 11,004 mg/L), 1 mM NEAA (non-essential amino acids), 100 μg/ml streptomycin and 100 U/ml penicillin in a humidified incubator (5% carbon-dioxide, 95% air) at 37°C as described previously [19].

### Treatments with Smokeless Tobacco Extract

Aqueous extracts of ST were prepared and standardized from batch to batch by measuring the nicotine content as described by us previously [19]. Cells (1000 cells/well) were plated in 96 well plates and allowed to grow for 24 h in complete medium. After 24 h, cells were treated with different concentrations (1-1000 μg/ml range) of ST for different time intervals 4, 12, 24, 48, 72 and 120 h, with STE replenished every 24 h. Cell viability was assessed using MTT assay as described earlier [19]. Cells were treated with ST (10 μg/ml, 48 hrs) [20,21] and harvested thereafter for western blotting/RT-PCR analysis/immunofluorescence.

#### Western blotting

Whole cell lysates were prepared from untreated (control) and ST-treated AMOL, HSC2 and SCC-4 cells. Protein concentrations were determined using Bradford reagent (Sigma) and equal amounts of proteins from untreated and treated cells were resolved on 10% sodium dodecyl sulphate (SDS)-polyacrylamide gels. The proteins were then electro-transferred onto polyvinylidenedifluoride (PVDF) membrane. After blocking with 10% non-fat milk in Tris-buffered saline (TBS, 0.1 M, pH = 7.4), blots were incubated with anti-PI Synthase antibody (a kind gift from Dr. Jackowski, St. JudeChildren's hospital, Tennesee, Memphis) or anti PI-3kinase or anti-cyclin D1 antibody (Santa Cruz Biotechnology Inc., Santacruz, CA) (dilution 1:200) at 4°C overnight. Protein abundance of α-tubulin served as a control for protein loading, and was determined with mouse monoclonal anti-α-tubulin antibody (Santa Cruz Biotechnology, CA). Membranes were incubated with secondary antibody, HRP-conjugated goat/mouse anti-IgG (Dako CYTOMATION, Denmark), at an appropriate dilution in 1% BSA, for 2 h at room temperature. After each step blots were washed thrice with Tween (0.1%)-Tris-buffered saline (TTBS). Protein bands were detected by enhanced chemiluminescence method (ECL, Amersham, Buckinghamshire, UK) on XO-MAT film.

#### Confocal laser scan microscopy

Cells (AMOL and HSC2) grown on coverslips were treated with ST (10 μg/ml, 48 hrs) in DMEM-F12 medium supplemented with 2% FBS at 37°C and processed for confocal laser scan microscopy as described by us [21,22].

#### Tissue specimens

This study was approved by Human Ethics Committee of All India Institute of Medical Sciences, New Delhi, India. For immunohistochemical analysis surgically resected tissues or biopsy specimens from OSCCs, OLs and normal oral tissues were obtained from Surgical Oncology Unit of Dr. B.R. Ambedkar Institute Rotary Cancer Hospital, All India Institute of Medical Sciences, New Delhi, India, with prior consent of the patients. The clinical and pathological data recorded included clinical tumor stage, site of the lesion, histopathological differentiation, age, gender and, tobacco and alcohol consumption habits in a pre-designed performa as described by us previously [23].

#### Clinicopathological characteristics of patients

Twenty one OSCC patients, ranging in age from 29 to 75 years were enrolled in this study. The diagnosis was based on clinical examination and histopathological analysis of the tissue specimens. The tumors were histologically graded as well, moderately or poorly differentiated SCCs. Twenty oral lesions were included in this study. The histopathological assessment scoring based on the architectural and cytological changes of epithelial dysplasia as described in the WHO classification and recently reviewed [24]. Oral lesions/leukoplakia (13 cases) were classified in two groups: (i) OLs with no dysplasia, (ii) OLs with dysplasia. Seven tissues taken from a distant site of OSCCs (with histologically confirmed normal oral epithelium hither to referred to as oral normal tissues) were also evaluated for PI Synthase protein expression.

### Tissue Microarray Immunohistochemistry

Oral squamous cancer Tissue Microarray (TMA) with 60 oral tissue sections (IMT-01250) was procured from Imgenex India Ltd (Bhubaneshwar, Orissa, India). Of the 60 oral tissue sections in TMA, 49 were OSCCs, 8 were oral lesions and 3 were non-malignant oral tissues. In addition, paraffin embedded sections (5 μm thickness) of human oral tissue specimens (21 OSCCs, 12 oral lesions and 7 non-malignant oral tissues) were stained with hematoxylin and eosin for histopathological analysis, and immunostaining was done on serial sections individually as described previously by Arora et al. [23]. The TMA slide and the individual tissue sections were de-paraffinized by baking at 62°C for 1 hour in vertical orientation and rehydrated prior to antigen retrieval. Thereafter, antigen retrieval was carried out using a microwave oven in 0.01 M-citrate buffer, pH 6.0. Endogenous peroxidase activity was blocked by incubating sections in methanol containing hydrogen peroxide (0.3%, v/v) for 20 min. Non-specific binding was blocked with 1% (w/v) BSA in PBS for 1 h and incubated with anti-PI Synthase antibody (1:200 dilution) for 16 h at 4°C. The primary antibody was detected using biotinylated secondary antibody and peroxidase labeled Streptavidin complex using Dako LSAB plus kit (Dako Labs, Glostrup, Denmark) and diaminobenzidine (DAB) as chromogen.

### Positive criterion for immunohistochemical staining

The immunopositive staining was evaluated in randomly selected five non-overlapping areas of the tissue sections with more than 80% epithelial cells. PI Synthase cytoplasmic immunoreactivity was classified into five categories, defined as follows: (0), l immunostaining in <10% cells; (+1), immunostaining in 10-30% cells; (+2), immunostaining in 30-50% cells; (+3), immunostaining in 50-70% cells and (+4), immunostaining in >70% of the cells showing cytoplasmic staining. Intensity was graded into four categories, defined as follows: (0), no detectable immunostaining or basal immunostaining; (+1) mild; (+2) moderate and (+3) intense immunostaining. The final score was calculated by adding the percentage positivity and intensity of staining scores (range 0-7). The immunohistochemical investigation was blind, i.e. the slides were coded and pathologist did not have prior knowledge of the local tumor burden, lymphonodular spread and grading of the OSCCs while scoring the immunoreactivity.

### Statistical analysis

The immunohistochemical data were subjected to statistical analysis using SPSS 10.0 software (Chicago). Box plot was prepared to determine the distribution of total score of PI Synthase protein expression in normal oral tissues, OLs and OSCCs. The performance of a diagnostic variable can be quantified by calculating the area under the ROC (receiver operating characteristic) curve. A graph of sensitivity against 1-specificity is called ROC curve. Sensitivity of a diagnostic test is the proportion of patients for whom the outcome is positive that is correctly identified by the test. Specificity is the proportion of patients for whom the outcome is negative that are correctly identified by the test. The ideal test would have an area under the ROC curve of 1, whereas a random guess would have an area under the ROC curve of 0.5. A better predictive power is attributed to a diagnostic variable as it approaches 1. ROC curve was plotted for PI Synthase for normal vs. OPLs, and normals Vs OSCCs using the final score obtained as described above. Sensitivity and specificity were calculated and quantified using receiver operating characteristic (ROC) analyses. Based on sensitivity and specificity values for PI Synthase, a cut-off ≥ 3 was defined as positive criterion for PI Synthase immunopositivity for statistical examination. The relationships between PI Synthase expression and clinicopathological parameters was tested in univariate analysis by Chi-Square and Fisher's exact test Two sided p-values were calculated and p ≤ 0.05 was considered to be significant.

## Results

To determine the biological significance of PI Synthase, we investigated the effect of ST on induction of this enzyme using oral cancer cell lines and oral epithelial cell cultures [19].

### Smokeless Tobacco activates PI Synthase in oral epithelial cell cultures

**Figure 1:**
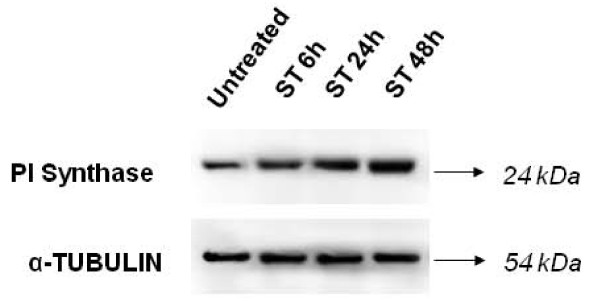
**Western blot analysis of PI Synthase in whole cell extracts of AMOL cells**. Cells were treated with 10 μg/ml of ST for varying time period. ST treated and untreated control cells protein extracts were resolved on SDS-PAGE gel, transferred on PVDF membrane and immunolabelled with PI synthase antibody, and developed using ECL as described in methodology section. Western blot of a control protein namely α-tubulin was used for comparison.

**Figure 2:**
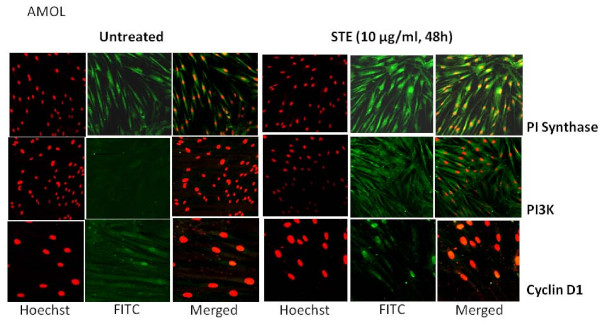
**Confocal micrographs showing the expression of PI Synthase, PI-3 Kinase and Cyclin D1 in AMOL cells after treatment with ST**. Cells grown on coverslips were treated with ST (10 μg/ml) for 48 h and immunolabelled with respective antibodies followed by FITC conjugated secondary antibody (Green fluorescence) and nuclei were counterstained with PI (red fluorescence). Original Magnification × 200.

**Figure 3:**
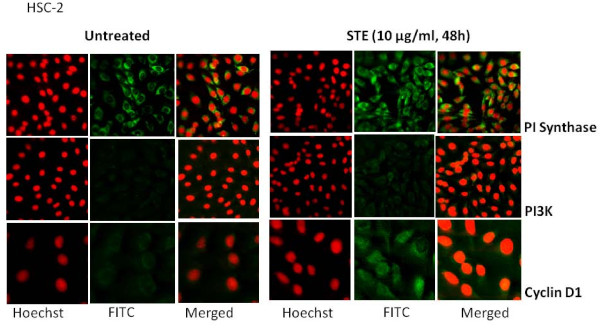
**Confocal micrographs showing the expression of PI Synthase, PI-3 Kinase and Cyclin D1 in HSC-2 cells after treatment with ST**. Cells grown on coverslips were treated with ST (10 μg/ml) for 48 h and immunolabelled with respective antibodies followed by FITC conjugated secondary antibody (Green fluorescence) and nuclei were counterstained with PI (red fluorescence). Original Magnification × 200.

To determine the optimal dose and duration for ST treatment, AMOL and HSC-2 cells were treated with different concentrations of ST ranging from 1 μg/ml to 1000 μg/ml for varying time periods ranging from 4 h to 120 h. Cell viability was measured by MTT assay. HSC-2 cells showed 25% increase in cell proliferation at 10 μg/ml concentration of ST after 48 h and 60% increase in cell proliferation at 10 μg/ml concentration of ST after 72 h. AMOL cells showed 20% increase in cell proliferation at 10 μg/ml concentration of ST after 48 h. Since higher doses were toxic for AMOL and HSC-2 cells, we chose 10 μg/ml of ST as the optimal dose for assessing the changes in protein expression levels upon enhanced cell proliferation. AMOL cells [19] were treated with 10 μg/ml of ST for varying time points. Untreated (control) AMOL cells expressed basal levels of PI Synthase (Figure 1). Treatment of AMOL cells with ST showed increase in PI Synthase expression within 6 h; while 2 fold enhanced expression was observed in 48 h (Figure 1). Hence, 10 μg/ml ST for 48 h was chosen for further experiments in oral cells. PI Synthase expression was analysed in AMOL and HSC-2 cells treated with ST (10 μg/ml) for 48 h by immunocytochemistry using a confocal microscope. Increased cytoplasmic expression of PI Synthase protein was observed in ST treated AMOL and HSC-2 cells as compared to the untreated control cells which expressed only basal levels of PI Synthase protein (Figures 2 and 3 respectively).

### Smokeless Tobacco induces PI3K and Cyclin D1 expression in oral cell cultures

Basal expression of PI3K was observed in both AMOL and HSC-2 cells (Figures 2 and 3 respectively). Treatment of cells with ST resulted in increased cytoplasmic expression of PI3-K protein (Figures 2 and 3 respectively) and cyclin D1 (Figures 2 and 3 respectively) in both cell types as compared to the untreated control cells, suggesting that ST induced PI Synthase expression, is accompanied by the induction of PI3-K and cyclin D1 in cultures established form OLs and OSCCs.

**Figure 4:**
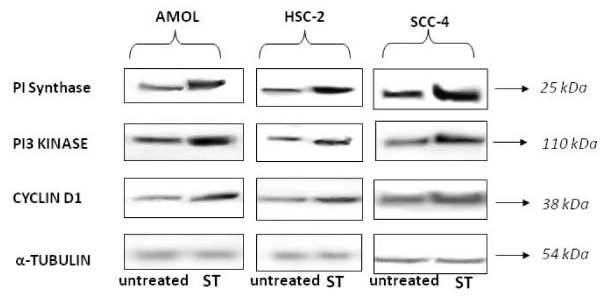
**Representative immunoblots using whole cell extracts for PI Synthase, PI3-Kinase and Cyclin D1 expression in AMOL, HSC-2 and SCC-4 cells**. ST treated (ST) and untreated control cells were immunolabelled with respective antibodies and developed using ECL. Western blot of a control protein namely α-tubulin was used for comparison.

The immunostaining findings were corroborated by western blot analysis: untreated AMOL, HSC-2 and SCC-4 cells expressed basal levels of PI Synthase protein (Figure 4, lane Untreated), while ST treatment resulted in increased expression of PI Synthase protein in AMOL (1.9 folds; Figure 4, lane ST), HSC-2 cells (2 folds; Figure 4, lane ST) and SCC-4 cells (2 folds; Figure 4, lane ST).

A 65 kDa band of PI3K protein was detected in cytoplasmic extracts of untreated AMOL, HSC-2 and SCC-4 cells (Figure 4). ST treatment increased the levels of PI3K protein in AMOL (1.8 folds), HSC-2 (2.1 folds) and SCC-4 (2 folds) cells (Figure 4). Further, exposure to ST resulted in increased expression of cyclin D1 protein in AMOL (2.4 folds), HSC-2 (2 folds) and SCC-4 cells (1.9 folds) as compared to the untreated control cells (Figure 4).

#### Tissue microarray (TMA) and Immunohistochemical analysis of PI Synthase expression in normal oral tissues, OLs and OSCCs and clinical correlations

**Figure 5:**
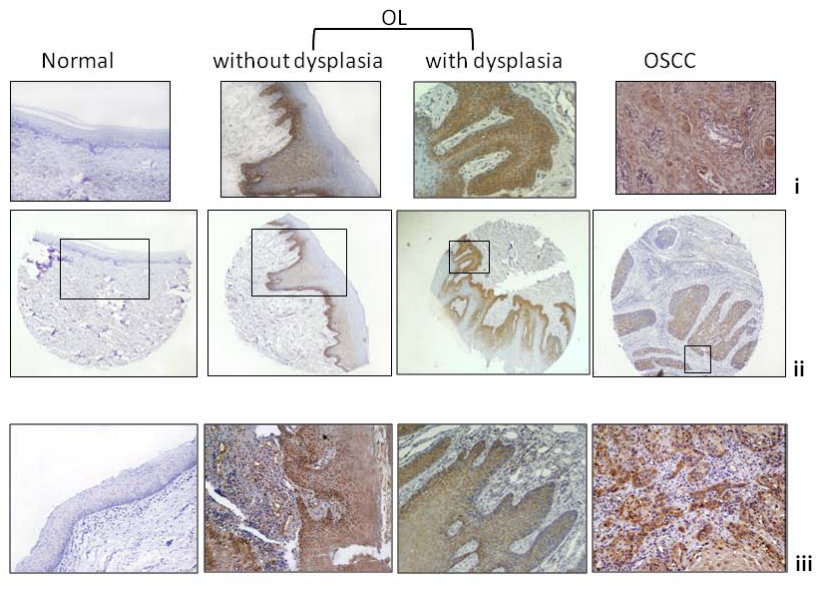
**Immunohistochemical analysis of PI Synthase protein in representative oral tissues and Tissue microarray sections i: Zoomed images of ii; ii: Tissue microarray sections; iii:Oral tissues**. Histologically normal tissue showing no detectable immunoreactivity; OLs tissue section without and with dysplasia depicting cytoplasmic staining; OSCC showing cytoplasmic staining.

Immunohistochemical analysis of PI Synthase was performed in individual sections of 21 OSCCs, 13 OLs and 7 histologically normal oral tissues. In addition, immunohistochemical analysis of PI Synthase was performed in OSCCs in a TMA format (Figure 4). Histologically proven normal oral tissue showed no detectable expression of PI Synthase protein (Figure 5). Cytoplasmic immunostaining was observed in OLs with no dysplasia (Figure 5). Intense cytoplasmic PI Synthase immunostaining was observed in dysplastic and OSCC lesions (Figure 5).

**Figure 6:**
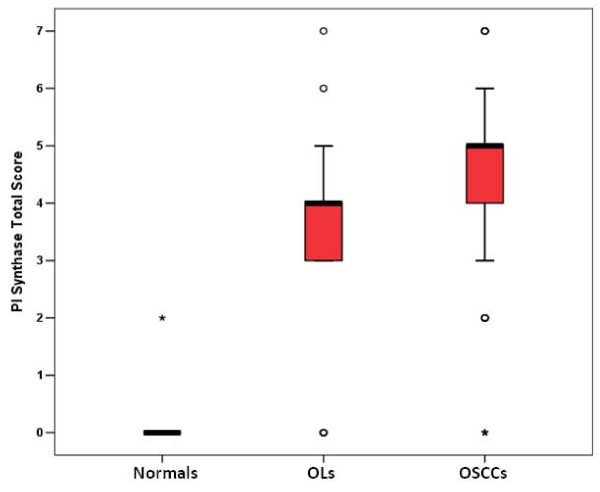
**Box-Plot analysis**. Box plots showing distribution of total immunostaining scores of PI Synthase protein determined by immunohistochemistry in paraffin-embedded sections of normal oral tissues, OLs and OSCCs. The vertical axis gives the total immunostaining score, obtained as described in the Methods section. Box plots showed increased expressions of PI Synthase in OSCCs with a median score (bold horizontal line) of 5 (range 3--6, as shown by vertical bars), as compared to non-malignant (histologically normal) oral tissues with a median score of 0.

**Figure 7:**
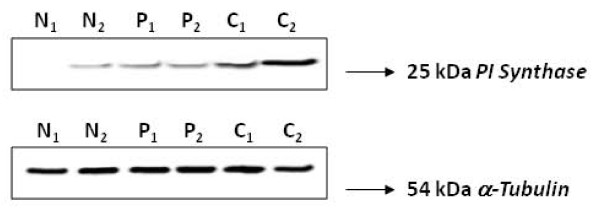
**Representative western blot for PI Synthase protein in oral tissues**. normal oral tissues (lane N1, N2); OPLs (lane P1, P2) and OSCCs (lane C1, C2) depicting α-tubulin used as a control protein

The results of immunohistochemical analysis of PI Synthase expression in normal oral tissues (10), OLs (20), and OSCCs (70); their correlation with clinicopathological parameters and tobacco consumption habits of the patients are summarized in Table 1. PI Synthase protein was localized predominantly in cytoplasm of epithelial cells. PI Synthase expression was observed in 16/20 (80%) OLs and 61/70 (87%) OSCCs. Increased expression of PI Synthase protein was observed in OLs and OSCCs as compared to normal (histologically non-malignant) oral tissues as shown in the Box plot analysis in Figure 6. Significant increase in PI Synthase expression was observed in OLs with no dysplasia, dysplasia and OSCC (p = 0.000). In OSCC patients, PI Synthase expression was significantly associated with tumor dedifferentiation (WDSCCs vs. MDSCCs and PDSCCs; p = 0.005) and tobacco consumption habits (p = 0.03). Figure 7 depicts 25-kDa band of PI Synthase protein in representative normal oral tissues, OLs and OSCCs obtained by western blot analysis.

**Table 1:** Analysis of PI Synthase protein expression in normal oral tissues, OLs and OSCCs: Correlation with clinicopathological parameters and tobacco consumption habits

Clinicopathological parameters	Total Cases	PI Synthase cytoplasmic positivity	
	N	n	(%)
**Normal Oral tissues (N)**	10	0	
			
**OLs**	20	16	80
**Differentiation**			
OLs w/o dysplasia	15	11	73
Dysplasia	5	5	100
Mild	2	2	
Moderate	2	2	
Severe	1	1	
**^Habits**			
**^Non Tobacco consumer**	2	2	
**^Tobacco consumer**	10	8	
^#^Tobacco chewer	9	8	
^#^Khaini (Tobacco with lime)	4	4	
^#^Gutkha	2	3	
^#^Betel Quid with tobacco	5	4	
^#^Tobacco smoking	4	4	
^#^Alcohol	3	3	
			
**OSCCs**	70	61	87
**Differentiation^a^**			
WDSCC	25	19	76
MDSCC	40	38	95
PDSCC	5	4	80
**^Habits^b^**			
**^Non Tobacco consumer**	4	2	
**^Tobacco consumer**	17	17	
^#^Tobacco chewer	16	5	
^#^Khaini (Tobacco with lime)	14	14	
^#^Gutkha	-	-	
^#^Betel Quid with tobacco	2	2	
^#^Tobacco smoking	4	4	
^#^Alcohol	4	3	

^#^Overlapping habits; ^cases excluding TMA

**a:**WDSCC Vs MDSCC + PDSCC (p = 0.005); **b**: tobacco consumer Vs non-consumer (p = 0.03, OR = 9.0)

#### Evaluation of PI Synthase as a potential biomarker

**Figure 8:**
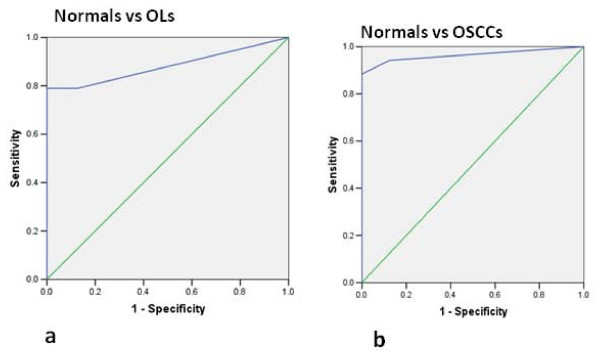
**Receiver operating characteristic curves of PI Synthase**.- a) normal vs OLs; b) normal vs OSCCs.

Receiver Operating Characteristic (ROC) analysis was used to determine the potential of PI Synthase as a biomarker for diagnosis of leukoplakia and OSCCs. The area-under-the-curve (AUC) values were 0.882 and 0.964 for normal versus OLs (Figure 8a) and OSCCs (Figure 8b), respectively indicating that PI Synthase may have the potential as a diagnostic biomarker for leukoplakia and OSCCs.

## Discussion

This study, to our knowledge, is the first report demonstrating overexpression of PI Synthase, PI3-K and cyclin D1 in ST treated cells from oral lesions and oral cancer. Analyzing clinical specimens from oral leukoplakic lesions without dysplasia, with dysplasia and OSCCs, we provide the first evidence of increased expression of PI Synthase in early stages of oral precancer and cancer and its correlation with tumor dedifferentiation and tobacco consumption.

High PI Synthase activity has been observed in *src- *and *erbB*2-transformed cells regulated by tyrosine kinase [25]; moreover, PI synthesis could be stimulated by addition of mitogenic growth factors and serum in quiescent normal rat kidney (NRK) cells. Herein, we demonstrated that in addition to these factors exposure to tobacco may be responsible for increased levels of PI Synthase. The biological and clinical significance of PI Synthase overexpression in human cancers remains unknown. However, PI Synthase has been shown to be essential for progression through G_1 _phase of cell cycle [26], mainly by increasing the cellular levels of cyclins D1 and E [27]. Previous studies [25,28,29] have shown that inhibitors of PI Synthase, inostamycin and δ-hexachloro-cyclohexane inhibit PI synthesis and blocked cell cycle progression in the G_1 _or S phase respectively. Inostamycin caused decrease in the cellular levels of cyclins D1 and E [25] and suppressed the invasion ability by reducing productions of matrix metalloproteinase-2 and -9 and cell motility in HSC-4 tongue carcinoma cell line [30].

**Figure 9:**
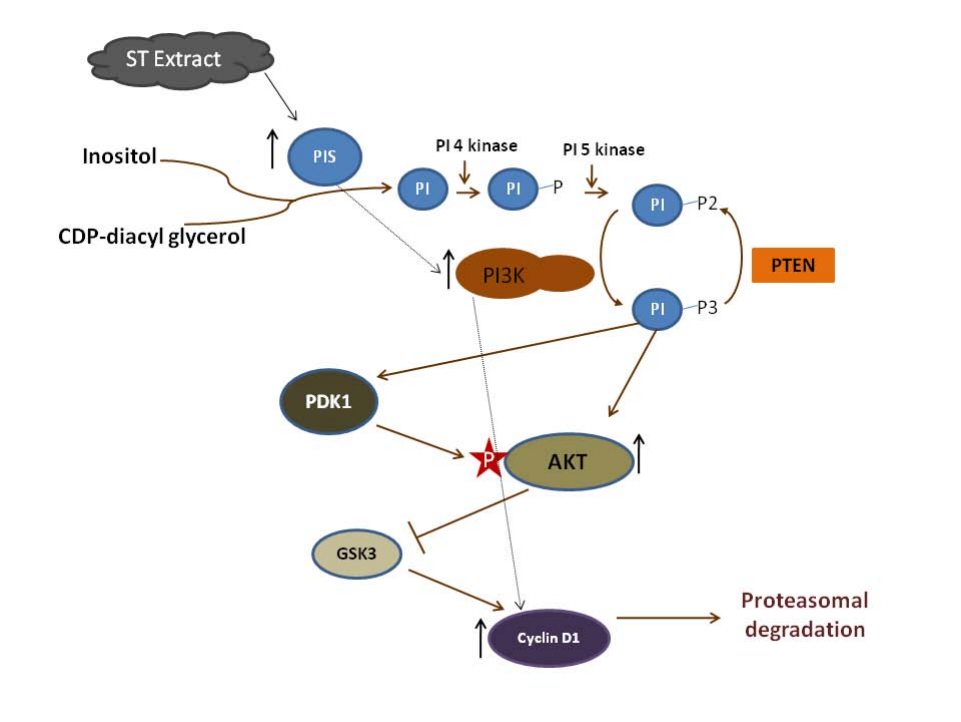
**Depicts the proposed signaling pathway on the basis of information accrued by the current study and available literature**. PIS: Phosphatidylinositol Synthase, PI3K: Phosphatidylinositol 3 Kinase, PI: Phospatidyinositol, PDK1: Phosphoinositide-Dependent Protein Kinase 1, GSK3: Glycogen Synthase Kinase 3, PTEN: Phosphatase and Tensin homolog, AKT: v-Akt Murine Thymoma Viral Oncogene Homolog 1

Hence, we propose that increased PI Synthase expression could play a role in cell transformation through the increased expression of cyclin D1, and possibly other growth-enhancing effects. PI Synthase increased the cellular levels of Akt kinase in serum-stimulated quiescent NIH3T3 cells, decreased doubling time and potentiated colony formation in soft agar [27]. Since the over expression of PI Synthase in NIH3T3 cells increased PI synthesis and the amounts of PIP-2 and PIP-3, it is possible that PI Synthase overexpression also enhances cellular levels of PIP-2 and PIP-3, which, together with serum, cause increased activation of Akt kinase. The PI3-K/Akt pathway not only regulates the transcriptional activity of cyclin D1, but also increases it accumulation by inactivating glycogen synthase kinase-3 (GSK3), which targets cyclin D1 for proteasomal degradation. Since PI Synthase overexpression enhances Akt kinase activation, this could explain the increased expression of the cyclin D1 protein. West et al. [31] showed that tobacco carcinogen (NNK) - induced cellular transformation and increased the activation of phosphatidylinositol 3'-kinase/Akt pathway in vitro and in vivo in lung tumorigenesis. Zheng et al. [32] demonstrated that nicotine induced PI3-K overexpression in lung cancer cells. Taken together, it suggests that upregulation of PI Synthase by ST may be one of the mechanisms by which ST exerts its effects on the PI-3 kinase pathway and other proteins involved in cellular proliferation such as cyclin D1 (Figure 9).

Immunohistochemical analysis of PI Synthase was performed in an oral cancer tissue microarray and few individual clinical samples of OLs, OSCCs and normal oral tissues. Significant increase in PI Synthase expression in lesions without dysplasia, dyplasias and OSCCs, suggests it to be an early event that occurs prior to development of malignancy. A salient finding of our study was the association of tobacco consumption with overexpression of PI Synthase in OSCCs. These clinical findings suggested that PI Synthase might be plausible molecular target of tobacco. Besides, PI3-K, has been implicated in the development and progression of numerous neoplasias including OSCCs [33,34]. Stahl et al. [35] suggested PI3-K as a marker of malignancy and tumor invasion and a potential target for pharmacological intervention in Head and Neck cancers.

## Conclusions

In **conclusion**, our in vitro studies showed increased PI Synthase, Cyclin D1 and PI3-K expression on exposure to ST in oral cell systems. In clinical samples, increased PI Synthase expression was found to be an early event in oral tumorigenesis, and is sustained during the development and progression of OSCC and is associated with tobacco consumption. These findings underscore its validation as a candidate diagnostic biomarker in oral cancer. Large-scale studies are warranted to further evaluate PI Synthase's potential as an indicator of progression risk in leukoplakia and a role in development and progression during early stages oral tumorigenesis.

## Abbreviations

MDSCC: Moderately Differentiated Squamous Cell Carcinoma; OPL: Oral Precancerous Lesion; OSCC: Oral Squamous Cell Carcinoma; PDSCC: Poorly Differentiated Squamous Cell Carcinoma; ST: Smokeless Tobacco; STE: Smokeless Tobacco Extract; TNF-alpha: Tumor Necrosis Factor-alpha; WDSCC: Well Differentiated Squamous Cell Carcinoma; DMEM: Dulbecco's modified Eagle medium.

## Competing interests

The authors declare that they have no competing interests.

## Authors' contributions

MS and JK both carried out the experimental work, data analysis and drafted the manuscript. study. MS, JK and SDG evaluated the H&E stained and immunostained slides. NKS and AS provided the clinical specimens for this study, clinical perspective and the follow-up data. RR conceived the study, participated in its design and coordination, provided infrastructural and financial support and edited the manuscript. All authors read and approved the final manuscript.

## Pre-publication history

The pre-publication history for this paper can be accessed here:

http://www.biomedcentral.com/1471-2407/10/168/prepub
